# Screening and Mechanism Study of Three Antagonistic Drugs, Oxysophoridine, Rutin, and Phellodendrine, against Zearalenone-Induced Reproductive Toxicity in Ovine Oocytes

**DOI:** 10.3390/antiox13060752

**Published:** 2024-06-20

**Authors:** Zongshuai Li, Tian Ma, Yali Liu, Wanruo Liu, Xingxu Zhao, Gaiping Zhang, Jianlin Wang, Yong Zhang

**Affiliations:** 1 College of Pastoral Agriculture Science and Technology, Lanzhou University, Lanzhou 730020, China; lizs@lzu.edu.cn; 2 State Key Laboratory of Grassland Agro Ecosystems, Lanzhou University, Lanzhou 730020, China; 3 Key Laboratory of Grassland Livestock Industry Innovation, Ministry of Agriculture and Rural Affairs, Lanzhou University, Lanzhou 730020, China; 4 Gansu Key Laboratory of Animal Generational Physiology and Reproductive Regulation, Gansu Agricultural University, Lanzhou 730070, China; mt13893610637@163.com (T.M.); lwr182311@163.com (W.L.); zhaoxx@gsau.edu.cn (X.Z.); 5 Second Hospital & Clinical Medical School, Lanzhou University, Lanzhou 730020, China; wo_lyl@163.com; 6 Longhu Laboratory of Advanced Immunology, Zhengzhou 450046, China; zhanggaip@126.com

**Keywords:** zearalenone, ovine oocytes, antioxidant drugs, oxysophoridine, rutin, phellodendrine, Smart-seq2

## Abstract

Zearalenone (ZEN) is a common fungal toxin with reproductive toxicity in various grains. It poses a serious threat to ovine and other animal husbandry industries, as well as human reproductive health. Therefore, investigating the mechanism of toxicity and screening antagonistic drugs are of great importance. In this study, based on the natural compound library and previous Smart-seq2 results, antioxidant and anti-apoptotic drugs were selected for screening as potential antagonistic drugs. Three natural plant compounds (oxysophoridine, rutin, and phellodendrine) were screened for their ability to counteract the reproductive toxicity of ZEN on ovine oocytes in vitro using quantitative polymerase chain reaction (qPCR) and reactive oxygen species detection. The compounds exhibited varying pharmacological effects, notably impacting the expression of antioxidant (*GPX*, *SOD1*, and *SOD2*), autophagic (*ATG3*, *ULK2*, and *LC3*), and apoptotic (*CAS3*, *CAS8*, and *CAS9*) genes. Oxysophoridine promoted *GPX*, *SOD1*, *ULK2*, and *LC3* expression, while inhibiting *CAS3* and *CAS8* expression. Rutin promoted *SOD2* and *ATG3* expression, and inhibited *CAS3* and *CAS9* expression. Phellodendrine promoted *SOD2* and *ATG3* expression, and inhibited *CAS9* expression. However, all compounds promoted the expression of genes related to cell cycle, spindle checkpoint, oocyte maturation, and cumulus expansion factors. Although the three drugs had different regulatory mechanisms in enhancing antioxidant capacity, enhancing autophagy, and inhibiting cell apoptosis, they all maintained a stable intracellular environment and a normal cell cycle, promoted oocyte maturation and release of cumulus expansion factors, and, ultimately, counteracted ZEN reproductive toxicity to promote the in vitro maturation of ovine oocytes. This study identified three drugs that antagonize the reproductive toxicity of ZEN on ovine oocytes, and compared their mechanisms of action, providing data support and a theoretical basis for their subsequent application in the ovine breeding industry, reducing losses in the breeding industry, screening of ZEN reproductive toxicity antagonists and various toxin antagonists, improving the study of ZEN reproductive toxicity mechanisms, and even protection of human reproductive health.

## 1. Introduction

Zearalenone (ZEN), one of the most common fungal toxins found in nature, was first isolated from moldy corn contaminated with *Fusarium graminearum* [1]. It is a non-steroidal estrogenic fungal toxin produced by *F. graminearum* and other *Fusarium* species as well as a potential endocrine disruptor, and therefore has strong reproductive toxicity to animals [2,3,4]. It has a stable structure, is not easily degradable, is widely distributed, can be present at any stage of crop processing, and easily contaminates various grains [4,5,6]. It poses a threat to the livestock breeding industry (pigs, sheep, and cows) [7,8,9,10], as well as human reproductive health. Therefore, it is of great importance to determine the mechanism of toxicity of ZEN and screen for effective antagonistic drugs. Previous research has shown that ZEN can cause cell cycle arrest in the G2/M phase, increase oxidative stress, disrupt spindle assembly, and impair mitochondrial activity in mouse oocytes by affecting the cell cycle, spindle assembly, oocyte activity, apoptosis, autophagy, and expression of genes related to primordial follicle assembly, ultimately damaging primordial follicle formation and oocyte maturation [11,12,13]. ZEN affects the meiotic maturation of porcine oocytes by inducing mitochondrial oxidative stress and cell apoptosis, and reducing endoplasmic reticulum stress levels, spindle cortex distribution, and autophagy [14,15]. ZEN causes damage to preantral follicles in ovine oocytes by participating in autophagy [16].

After efforts using chemical (e.g., ammonia, hydrogen peroxide, and sodium hypochlorite), physical (e.g., adsorption and extraction through modified montmorillonite), and biological (e.g., bread yeast cell wall adsorption and *Bacillus subtilis* degradation) methods to reduce the reproductive toxicity of ZEN to animals and humans, safe natural compounds have emerged as a new research focus [17,18,19]. Research has shown that betulinic acid can alleviate ZEN injury to the mouse uterus by inhibiting the activity of catalase (CAT) and superoxide dismutase (SOD) [20]. Furthermore, lycopene reportedly mitigates ZEN-induced damage to the mouse uterus by alleviating hormonal imbalances, such as estradiol and progesterone, enhancing antioxidant capacity, and reducing inflammatory factor expression [21]. Additionally, resveratrol has been shown to correct mitochondrial depolarization, oxidative stress, and apoptosis through the PINK1/Parkin pathway, thereby reducing the damage caused by ZEN to porcine oocytes [14]. Finally, isorhamnetin has been shown to stimulate *SOD2* protein expression and inhibit cell apoptosis through the PI3K/Akt signaling pathway, thereby reducing the damage caused by ZEN to porcine oocytes [22]. Agricultural breeding livestock are sensitive to ZEN; however, there is a lack of investigation into screening potential drugs that could reduce the reproductive toxicity of ZEN in ovines.

The combination of Smart-seq2 technology and preliminary results of this study showed that ZEN could increase oxidative stress, interfere with the spindle assembly and cell autophagy, and ultimately inhibit ovine oocyte maturation by affecting the expression of genes related to apoptosis, oxidative stress, autophagy, oocyte maturation, and spindle assembly [10]. Therefore, a constantly updated natural plant compound library and the screening of antagonistic drugs were combined in this study, focusing on two types of substances: antioxidants and autophagy inhibitors. The aims of this study were to provide a theoretical basis and reference data for further studies on the reproductive toxicity mechanism of ZEN, screening drugs that antagonize reproductive toxicity of ZEN in ovines, reducing losses in the breeding industry, and even providing a reference for protecting human reproductive health.

## 2. Materials and Methods

### 2.1. Smart-Seq2 Omics Data Analysis and Candidate Drug Identification

By analyzing previous Smart-seq2 data and consulting the literature, we screened seven gene sets comprising differential genes involved in oocyte maturation, cumulus expansion factor, spindle checkpoint, cell apoptosis, autophagy, and oxidative stress. Employing STRING (https://cn.string-db.org/; accessed on 4 April 2024 to 23 May 2024) online software and Cytoscape (version: 3.3.0) software, with sheep as a reference species, we obtained protein-protein interaction (PPI) network diagrams corresponding to the seven selected gene sets. Finally, based on PPI data, we determined the types and quantities of candidate drugs.

In the preliminary experiment, Smart-seq2 sequencing samples included only the control group (without ZEN; C) and the experimental group (with ZEN; T), each with three replicates. Replicates in the control group were labeled C1, C2, and C3, whereas replicates in the processing group were labeled T1, T2, and T3. Heatmaps of the Smart-seq2 data for the seven mentioned gene sets were generated. The treatment methods for the blank group (untreated; CK) and control group (with ZEN; ZEN) in this study were identical to those used in the previous experiment for the control (C) and experimental (T) groups. We cross-validated the qPCR results of the genes tested in this study in the CK and ZEN groups with the Smart-seq2 data results from previous experiments, thereby confirming the accuracy of the two experiments.

### 2.2. In Vitro Culture of Ovine Oocytes

Washing and maturation solutions for ovine oocytes were prepared before the experiment. The washing solution (40 mL) consists of 39 mL of M199 (Biological Industries, Kibbutz Beit Haemek, Israel), 1 mL of HEPES (1M) (Sigma Aldrich, St. Louis, MO, USA), and 160 mg of bovine serum albumen (BSA; Sigma Aldrich). The maturation solution (10 mL) is composed of 10 mL M199 (Biological Industries), 10 µL follicle stimulating hormone (FSH) (5 μg/mL; Sigma Aldrich), 10 µL luteinizing hormone (LH) (5 μg/mL; Sigma Aldrich), 10 µL estradiol (1 μg/mL; Sigma Aldrich), 10 µL dual antibody (100 μg/mL; Biological Industries), and 40 mg BSA (Sigma Aldrich).

Samples were collected from an ovine slaughterhouse. After slaughter, the ovaries were removed, placed in sterile phosphate-buffered saline (PBS) (Biological Industries) containing penicillin and streptomycin (100 μg/mL; Biological Industries) at 37 °C, and transported back to the laboratory within 3 h. The ovaries were washed 2–3 times with sterile PBS, immersed in PBS containing dual antibodies (100 μg/mL; Biological Industries), and placed in a 37 °C water bath for later use.

Oocytes wash solution (15 mL) was transferred to a 37 °C water bath using a sterile centrifuge tube. Subsequently, 60- and 35-mm cell culture dishes were prepared. A 3 mm × 3 mm square was drawn at the bottom, 5 mL and 2.5 mL of oocyte wash solution was added, respectively, and the dishes were placed in the cell culture incubator for later use. Follicular fluid was carefully aspirated using a 5 mL syringe and injected into a centrifuge tube containing the oocyte wash solution. The bottom liquid was gently removed when picking up oocytes and placed in the 60 mm culture dish. Under the microscope, the cumulus oocyte complex (COC) was transferred into a 35 mm culture dish (this procedure was repeated once). Finally, it was transferred into the mature liquid and a 5% CO_2_ and 37 °C incubator for cultivation.

COCs with a uniform cytoplasm and three or more layers of dense cumulus cells (Grade A) and one to three layers of cumulus cells (Grade B) around the periphery were selected.

### 2.3. Preliminary Screening of Drugs and Selection of Working Concentrations

Nine compounds—three natural autophagy inhibitors (diosgenin glucoside, syringin, and liensinine) (TargetMol, Shanghai, China) and six antioxidants (phellodendrine, oxysophoridine, isolongifolene, L-ascorbic acid, morroniside, and rutin) (TargetMol, Shanghai, China)—were selected for this study. Due to the non-diffusion of oocytes, CCK8 determination was not performed during the initial screening of the nine drugs, and their initial addition amount was set at 10^−6^ mol/L. Experiments included a blank group (untreated; CK), control group (with ZEN), and treatment group (with ZEN and natural products; named according to actual conditions). Subsequent experiments will also follow this naming convention. 

After 36 h of in vitro culture (5% CO_2_ and 37 °C), granulosa cells were removed with hyaluronidase and the naked oocytes were transferred into a centrifuge tube containing PBS. After centrifugation at 1200 rpm/min for 5 min, the supernatant was discarded and TRIzoL (350 µL) (Thermo Fisher Scientific, Waltham, MA, USA) was added for lysis. Total RNA was extracted from samples using an RNA extraction kit (OMEGA, Norcross, Georgia, USA). cDNA was obtained using a reverse transcription kit (Thermo Fisher Scientific). A QuantiNova SYBR Green PCR Kit (Qiagen, Dusseldorf, Germany) was used for qPCR assays. qPCR was used to detect the levels of expression of *BMP15*, *CDC20*, and *GDF9*, genes related to oocyte maturation, to preliminarily screen natural compounds (Table 1).

Specific primers were designed using the NCBI Primer-BLAST tool to amplify fragments corresponding to the selected genes. All samples were amplified in triplicate, and the mean and standard error values were calculated. Relative to *GAPDH*, expression levels of all genes were calculated using the 2^−ΔΔCT^ method; *p* < 0.01 is extremely significant, *p* < 0.05 is significant, while genes with a trend of change but not significant have not been labeled.

The final concentration of ZEN and drugs, the number of experimental replicates, number of oocytes per time period, and first polar body excretion rate are shown in Table 2. The exclusion rate of the first polar body was analyzed for variance using SPSS 19 and multiple comparisons were tested, thus obtaining the maturation rate of oocytes.

After preliminary screening to obtain candidate effective natural drugs, concentration gradient tests were conducted on candidate drugs to determine the optimal working concentration. A blank group, control group, and treatment group were established (design gradient). The concentrations of natural products added to the treatment groups were 10^−4^, 10^−5^, 10^−6^, 10^−7^, and 10^−8^ mol/L (Table 3). After 36 h of in vitro culture (5% CO_2_ and 37 °C), cDNA was isolated, as previously described. Similarly, the gene expression levels of BMP15, CDC20, and GDF9 were determined using qPCR to determine optimal working concentrations (Table 1). Simultaneously, we calculated the first polar body exclusion rate for each group (Table 3).

### 2.4. qPCR Detection of Gene Expression

After determining the final type and working concentration of natural compounds, blank, control, and treatment groups were established (Table 4). After 36 h of in vitro culture (5% CO_2_ and 37 °C), cDNA was obtained according to the previously described method and genes related to oxidative stress, cell apoptosis, autophagy, oocyte maturation, cumulus expansion factor, cell cycle, and spindle assembly were detected using qPCR (Table 1). Simultaneously, we calculated the first polar body exclusion rate for each group (Table 4).

### 2.5. Reactive Oxygen Species Immunofluorescence Detection

Blank, control, and treatment groups were established. After 36 h of in vitro culture (5% CO_2_ and 37 °C), naked oocytes were collected according to the method above for future use. Each group was stained according to the instructions of the reactive oxygen species (ROS) staining kit (BioVision, San Francisco Bay, CA, USA), and images were captured.

## 3. Results

### 3.1. Preliminary Screening of Drugs

**Figure 1:**
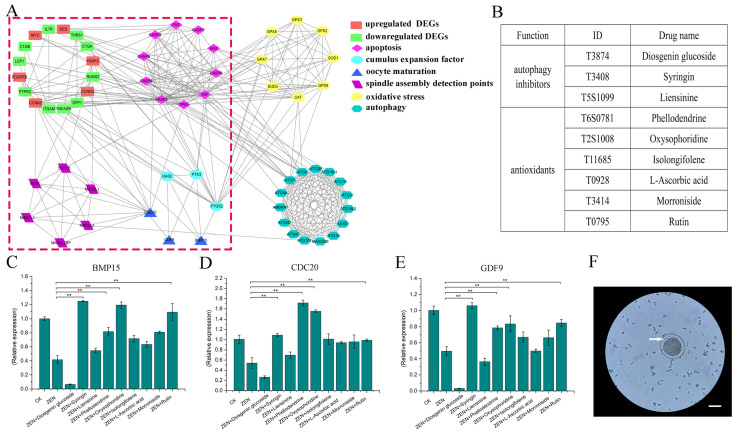
Preliminary screening of drugs. (**A**) PPI analysis network diagram corresponding to transcriptome data, (**B**) the names and corresponding product IDs of nine drugs, (**C**–**E**) qPCR results of *BMP15*, *CDC20*, and *GDF9* for each group (**, *p* < 0.01 is extremely significant; while genes with a trend of change but not significant have not been labeled), (**F**) the arrow points to the polar body (10×, 100 μm).

The preliminary omics results indicate that the four gene sets, namely apoptosis, oocyte maturation, oocyte amplification, and spindle monitoring points, are closely related to differentially expressed genes (DEGs) in omics, with the apoptosis gene set occupying a core position (Figure 1A, within the red box). Further analysis revealed that the gene sets for autophagy and oxidative stress are closely related to cell apoptosis, and the oxidative stress gene set also affects diffusion-related genes of cumulus cells (Figure 1A). This suggests that regulating autophagy and oxidative stress in oocytes may counteract ZEN-induced cell damage. Therefore, six antioxidants and three autophagy inhibitors were selected as candidate drugs (Figure 1B). ZEN can inhibit the expression of oocyte maturation-related genes (*BMP15*, *CDC20*, and *GDF9*). Based on the results of candidate drugs offsetting the inhibitory effect of ZEN, syringin, oxysophoridine, rutin, and phellodendrine were initially selected for subsequent experiments (Figure 1C–E). The first polar body (Figure 1F) exclusion rate of each group is shown in Table 2, which was consistent with the qPCR results.

### 3.2. Concentration Determination of the Selected Drugs

**Figure 2:**
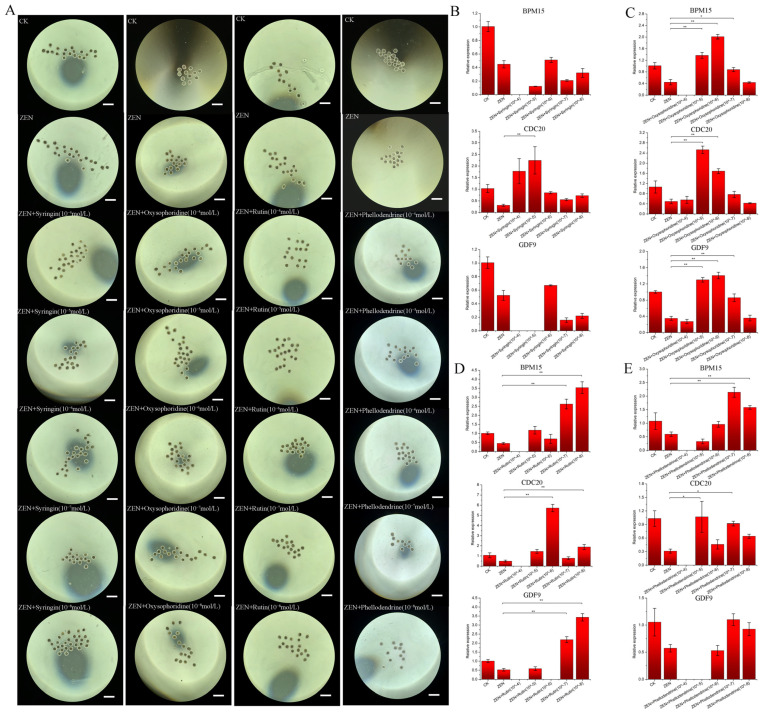
Concentration determination of the selected drugs. (**A**) Each of the four drugs is set up in seven groups, the growth status of sheep oocytes cultured in vitro after 36 h of exposure to each group (CK, ZEN, ZEN + drug (10^−4^ mol/L), ZEN + drug (10^−5^ mol/L), ZEN + drug (10^−6^ mol/L), ZEN + drug (10^−7^ mol/L), ZEN + drug (10^−8^ mol/L)) (4×, 250 μm), with ZEN concentration of 20 μM, (**B**) qPCR results of *BMP15*, *CDC20*, and *GDF9* in the seven groups set up in syringin, (**C**) qPCR results of *BMP15*, *CDC20*, and *GDF9* in the seven groups set up in oxysophoridine, (**D**) qPCR results of *BMP15*, *CDC20*, and *GDF9* in the seven groups set up in rutin, (**E**) qPCR results of *BMP15*, *CDC20*, and *GDF9* in the seven groups set up in phellodendrine. (**, *p* < 0.01 is extremely significant; *, *p* < 0.05 is significant; while genes with a trend of change but not significant have not been labeled).

Based on preliminary screening results, gradient settings were applied to the four drugs for further screening. When collecting naked oocytes, the number of each group ranged from 15 to 21 (Figure 2A). The use of qPCR to detect the expression of *BMP15*, *CDC20*, and *GDF9* in each concentration group showed that the efficacy of Syringin was not ideal (Figure 2B). The reason for the unstable effect of Syringin may be due to batch effects of oocytes or other unknown reasons. To ensure the stability of the therapeutic effect, this drug will not be considered in subsequent trials. Optimal working concentrations for the antioxidant drugs, oxysophoridine, rutin, and phellodendrine, were determined to be 10^−6^, 10^−8^, and 10^−7^ mol/L, respectively (Figure 2C–E). At the corresponding concentrations, the expression levels of mature genes in the three oocytes were increased significantly (Figure 2C–E). The trend in the first polar body excretion rate was basically consistent with the qPCR results (Table 3), but it was not more pronounced than the qPCR results due to batch effects and other reasons.

### 3.3. Detection of Genes Related to Oocyte Maturation

**Figure 3:**
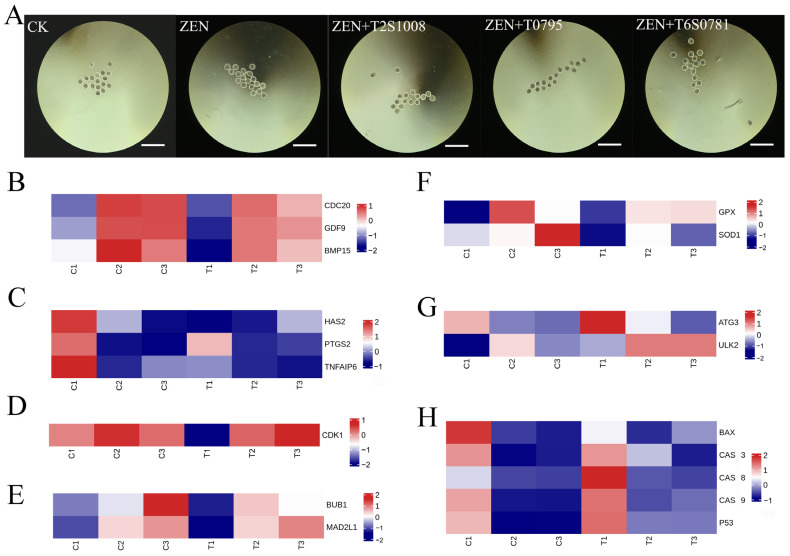
Oocyte status at optimal working concentration and heat maps of omics data for each gene set. (**A**) The growth status of sheep oocytes cultured in vitro after 36 h of exposure to five groups (CK, ZEN, ZEN + oxysophoridine (10^−6^ mol/L), ZEN + rutin (10^−8^ mol/L), ZEN + phellodendrine (10^−7^ mol/L)) (4×, 250 μm), with ZEN concentration of 20 μM/L, (**B**) heat map of genes related to oocyte maturation, (**C**) heat map of genes related to cumulus expansion factors, (**D**) heat map of genes related to cell cycle, (**E**) heat map of genes related to spindle checkpoint, (**F**) heat map of genes related to oxidative stress, (**G**) heat map of genes related to autophagy, (**H**) heat map of genes related to apoptosis.

**Figure 4:**
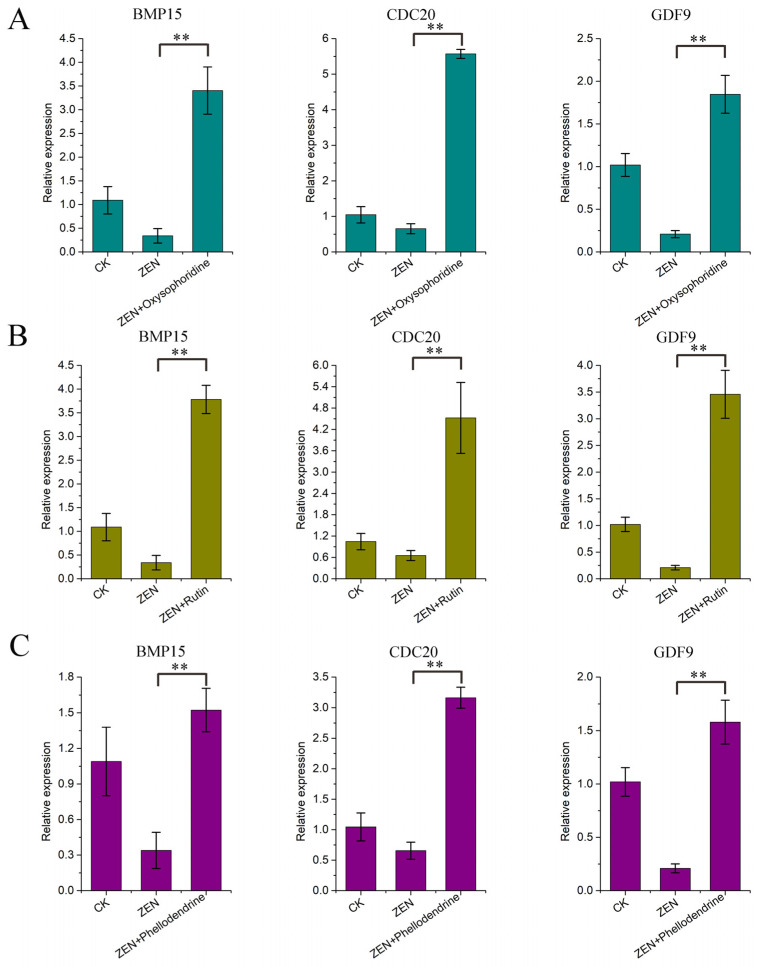
qPCR results of genes associated with oocyte maturation. (**A**) qPCR results corresponding to *BMP15*, *CDC20*, and *GDF9* of oxysophoridine, (**B**) qPCR results corresponding to *BMP15*, *CDC20*, and *GDF9* of rutin, (**C**) qPCR results corresponding to *BMP15*, *CDC20*, and *GDF9* of phellodendrine (**, *p* < 0.01 is extremely significant; while genes with a trend of change but not significant have not been labeled).

Subsequent experiments and associated gene testing were conducted at the optimal working concentrations of the three drugs, and the number of naked oocytes collected in each group ranged from 15 to 21 (Figure 3A). The first polar body excretion rate indicated that all three drugs can significantly improve the maturation rate of oocytes under ZEN treatment (Table 4). The qPCR results for *BMP15*, *CDC20*, and *GDF9* showed that ZEN inhibited the expression of the three genes, which is consistent with the omics results (Figure 3B). Oxysophoridine and rutin promoted the expression of the three genes significantly (*p* < 0.01) (Figure 4A,B), phellodendrine also promoted the expression of the three genes significantly (BMP15, *p* < 0.05; CDC20 and GDF9, *p* < 0.01) (Figure 4C), offsetting the ZEN inhibitory effect.

### 3.4. Expression of Genes Related to Cumulus Expansion Factors

**Figure 5:**
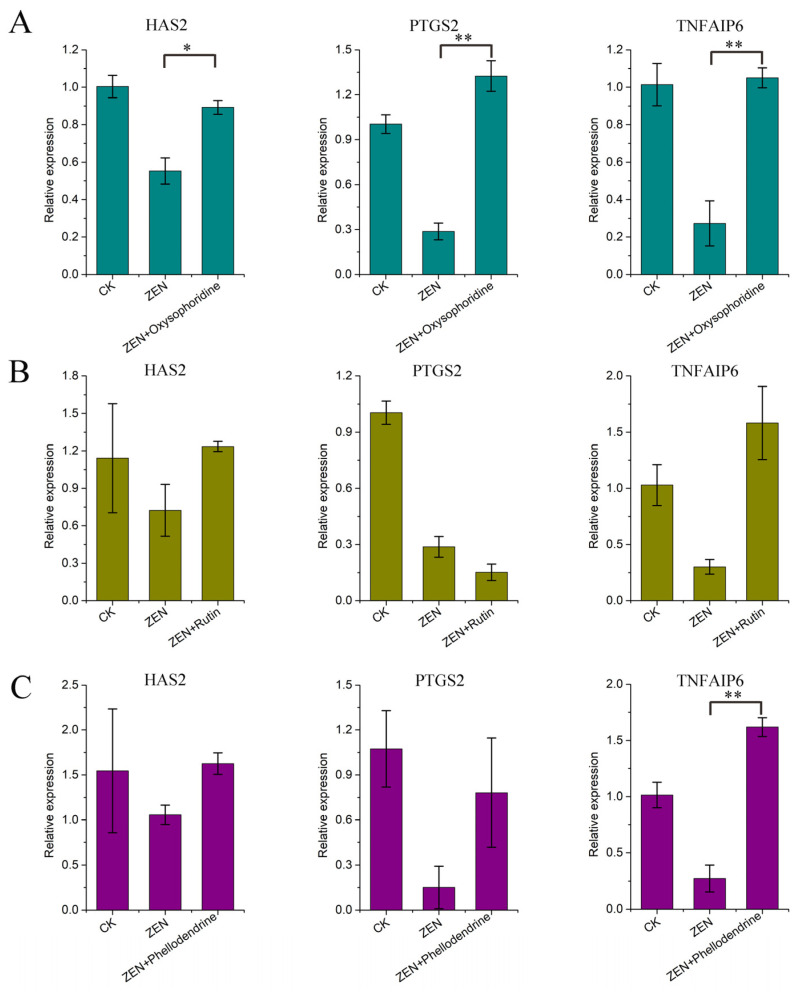
qPCR results of genes related to cumulus expansion factors. (**A**) qPCR results corresponding to *HAS2*, *PTGS2*, and *TNFAIP6* of oxysophoridine, (**B**) qPCR results corresponding to *HAS2*, *PTGS2*, and *TNFAIP6* of rutin, (**C**) qPCR results corresponding to *HAS2*, *PTGS2*, and *TNFAIP6* of phellodendrine (**, *p* < 0.01 is extremely significant; *, *p* < 0.05 is significant; while genes with a trend of change but not significant have not been labeled).

The qPCR results for *HAS2*, *PTGS2*, and *TNFAIP6* showed that ZEN inhibited their expression, which is consistent with our omics data (Figure 3C). Oxysophoridine promoted the expression of three genes significantly (*HAS2*, *p* < 0.05; *PTGS2* and *TNFAIP6*, *p* < 0.01) (Figure 5A). Rutin has a good promotion effect on *HAS2* and *TNFAIP6*; however, it inhibits *PTGS2* expression (Figure 5B). Phellodendrine had a promotive effect on all three genes, and has a significant effect on *TNFAIP6* (*p* < 0.01) (Figure 5C).

### 3.5. Expression of Cell Cycle-Related Genes

**Figure 6:**
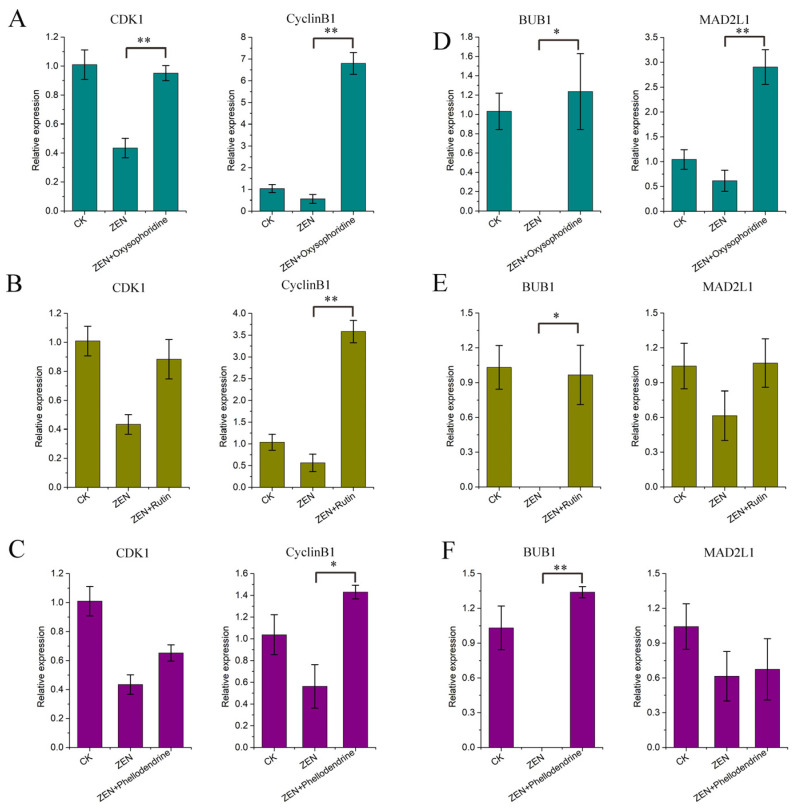
qPCR results of cell cycle-related genes and spindle checkpoint-related genes. (**A**) qPCR results corresponding to *CDK1* and *CyclinB1* of oxysophoridine, (**B**) qPCR results corresponding to *CDK1* and *CyclinB1* of rutin, (**C**) qPCR results corresponding to *CDK1* and *CyclinB1* of phellodendrine, (**D**) qPCR results corresponding to *BUB1* and *MAD2L1* of oxysophoridine, (**E**) qPCR results corresponding to *BUB1* and *MAD2L1* of rutin, (**F**) qPCR results corresponding to *BUB1* and *MAD2L1* of phellodendrine (**, *p* < 0.01 is extremely significant; *, *p* < 0.05 is significant; while genes with a trend of change but not significant have not been labeled).

The qPCR results for *CDK1* and *CyclinB1* showed that ZEN inhibited the expression of both genes, which is consistent with our omics data (Figure 3D). Oxysophoridine significantly promoted the expression of two genes significantly (*p* < 0.01) (Figure 6A). In addition, rutin and phellodendrine had a good promotive effect on two genes, and a significant promotive effect on *TNFAIP6* (*p* < 0.01) (Figure 6B,C).

### 3.6. Expression of Spindle Checkpoint-Related Genes

The qPCR results for *BUB1* and *MAD2L1* showed that ZEN inhibited their expression, which is consistent with the omics results (Figure 3E). Oxysophoridine promoted the expression of two genes significantly (*BUB1*, *p* < 0.05; *MAD2L1*, *p* < 0.01) (Figure 6D). In addition, rutin had a good promotive effect on the two genes, and a significant promotive effect on *BUB1* (*p* < 0.05) (Figure 6E). Furthermore, phellodendrine had a good promotive effect on the two genes, and a significant promotive effect on *BUB1* (*p* < 0.01) (Figure 6F).

### 3.7. Expression of Oxidative Stress-Related Genes

**Figure 7:**
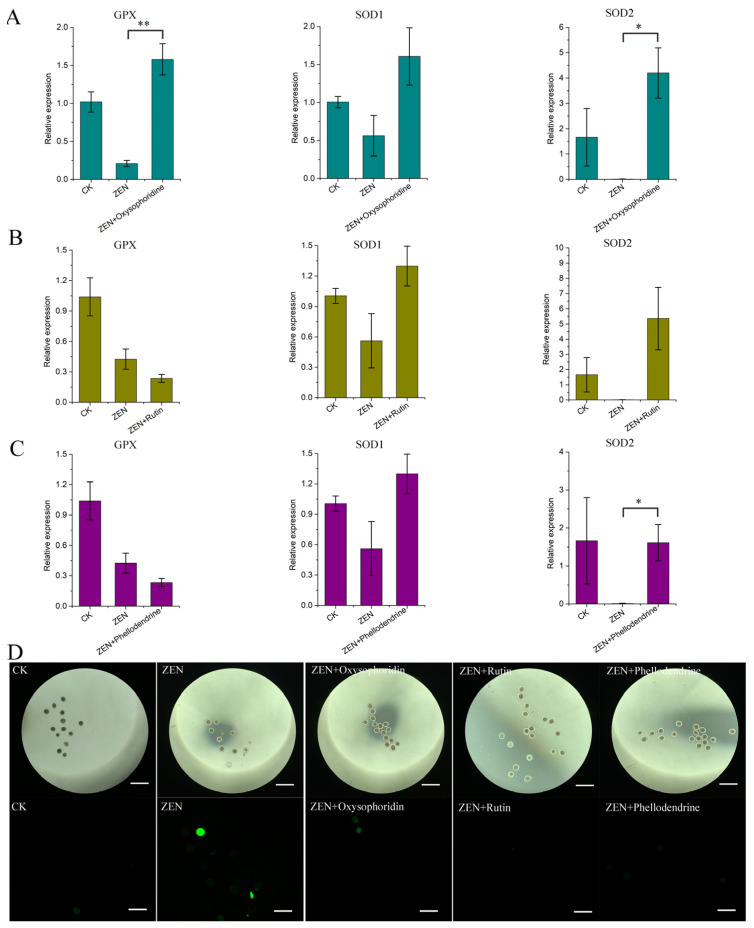
qPCR results of oxidative stress-related genes. (**A**) qPCR results corresponding to *GPX*, *SOD1*, and *SOD2* of oxysophoridine, (**B**) qPCR results corresponding to *GPX*, *SOD1*, and *SOD2* of rutin, (**C**) qPCR results corresponding to *GPX*, *SOD1*, and *SOD2* of phellodendrine, (**D**) white light and ROS staining results of oocytes in groups CK, ZEN, ZEN + oxysophoridine, ZEN + rutin and ZEN+ phellodendrine (**, *p* < 0.01 is extremely significant; *, *p* < 0.05 is significant; while genes with a trend of change but not significant have not been labeled) (4×, 250 μm).

The qPCR results for *GPX*, *SOD1*, and *SOD2* showed that ZEN inhibited the expression of these three genes, which is consistent with the omics data (Figure 3F). Oxysophoridine had a good promotive effect on three genes, and significant promotive effects on GPX (*p* < 0.01) and SOD2 (*p* < 0.05) (Figure 7A). Rutin had a good promotive effect on *SOD1* and *SOD2*, but it inhibited *GPX* expression (Figure 7B). Phellodendrine had a good promotive effect on *SOD1* (*p* < 0.05) and *SOD2*, but it inhibited *GPX* expression (Figure 7C). ROS immunofluorescence detection showed that all three drugs reduced the accumulation of ROS in oocytes substantially (Figure 7D).

### 3.8. Expression of Autophagy-Related Genes

**Figure 8:**
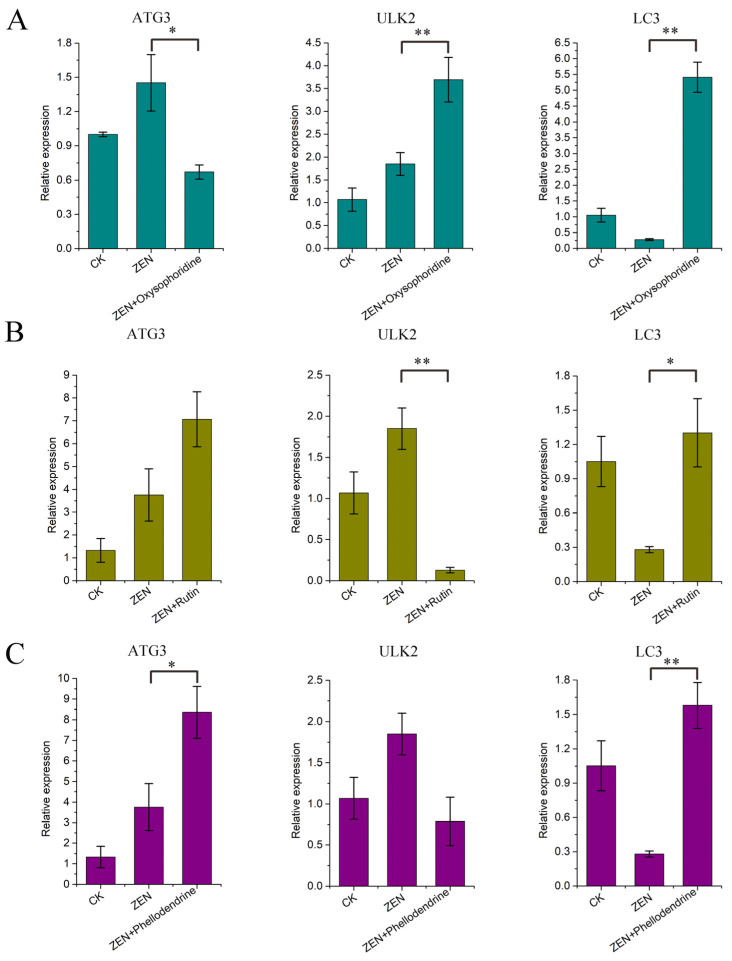
qPCR results of autophagy-related gene expression. (**A**) qPCR results corresponding to *ATG3*, *LC3*, and *ULK2* of oxysophoridine, (**B**) qPCR results corresponding to *ATG3*, *LC3*, and *ULK2* of rutin, (**C**) qPCR results corresponding to *ATG3*, *LC3*, and *ULK2* of phellodendrine (**, *p* < 0.01 is extremely significant; *, *p* < 0.05 is significant; while genes with a trend of change but not significant have not been labeled).

The qPCR results for *ATG3*, *LC3*, and *ULK2* showed that ZEN promoted the expression of *ATG3* and *ULK2* while inhibiting the expression of *LC3* (no omics data available), which is consistent with the omics data (Figure 3G). Oxysophoridine promoted the expression of three genes significantly (*ATG3*, *p* < 0.05; *LC3* and *ULK2*, *p* < 0.01) (Figure 8A). Rutin had a good promotive effect on *ATG3* and *LC3* (*p* < 0.05), but it inhibited the expression of *ULK2* (*p* < 0.01) (Figure 8B). Phellodendrine had a significant promotive effect on *ATG3* (*p* < 0.05) and *LC3* (*p* < 0.01), but it inhibited *ULK2* expression (Figure 8C).

### 3.9. Expression of Apoptosis-Related Genes

**Figure 9:**
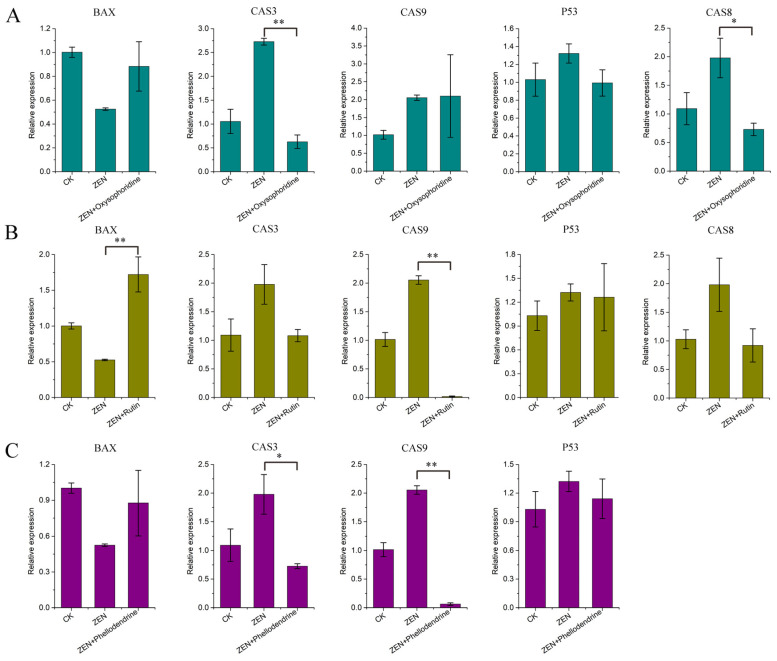
qPCR results of apoptosis-related genes. (**A**) qPCR results corresponding to *BAX*, *CAS3*, *CAS8*, *CAS9*, and *P53* of oxysophoridine, (**B**) qPCR results corresponding to *BAX*, *CAS3*, *CAS8*, *CAS9*, and *P53* of rutin, (**C**) qPCR results corresponding to *BAX*, *CAS3*, *CAS9*, and *P53* of phellodendrine (**, *p* < 0.01 is extremely significant; *, *p* < 0.05 is significant; while genes with a trend of change but not significant have not been labeled).

The qPCR results for *BAX*, *CAS3*, *CAS8*, *CAS9*, and *P53* showed that ZEN promoted the expression of *CAS3*, *CAS8*, *CAS9*, and *P53* and inhibited the expression of *BAX*, which is consistent with the omics data (Figure 3H). Oxysophoridine inhibited *CAS3* (*p* < 0.01) and *CAS8* (*p* < 0.05) expression significantly, inhibited *P53* expression, had minimal effect on *CAS9*, but promoted *BAX* expression (Figure 9A). Rutin had good inhibitory effects on *CAS3*, *CAS8*, *CAS9* (*p* < 0.01), and *P53*, but significantly promoted *BAX* expression (*p* < 0.01) (Figure 9B). Phellodendrine had a good inhibitory effect on *CAS3* (*p* < 0.05), *CAS9* (*p* < 0.01), and *P53*, but promoted *BAX* expression, whereas *CAS8* was not detected (Figure 9C).

### 3.10. Diagram of the Mechanism of Three Drugs Reducing the Reproductive Toxicity of ZEN on Sheep Oocyte IVM

**Figure 10:**
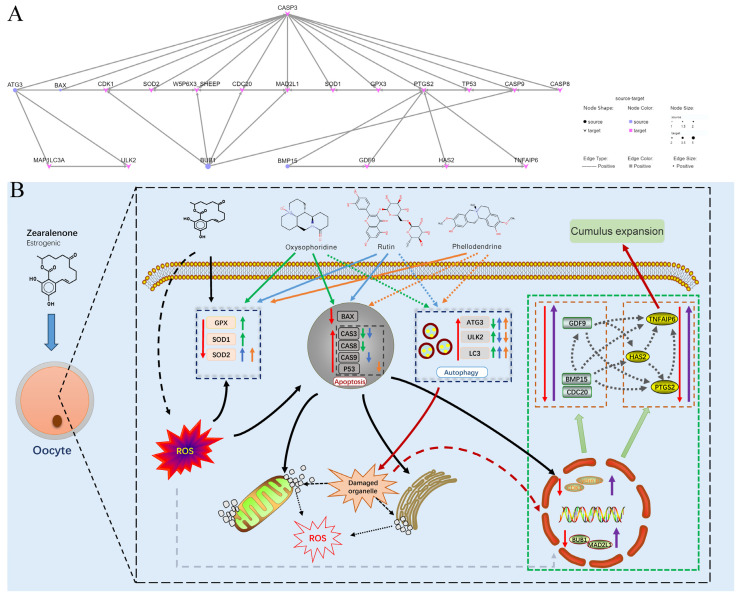
Diagram of the mechanism of three drugs reducing the reproductive toxicity of ZEN on sheep oocyte IVM.

Based on the directed network of genes corresponding to proteins (Figure 10A), we inferred the mechanism network diagram of three drugs reducing the reproductive toxicity of ZEN on ovine oocyte IVM through antioxidant effects and inhibition of cell apoptosis (Figure 10B).

## 4. Discussion

ZEN, as a potential endocrine disruptor toxin [23], has reproductive toxicity to both female and male animals [23,24] but its effect is more pronounced in females. This toxin can exert its effects through various pathways such as oxidative stress, cell apoptosis, and DNA damage, causing reproductive damage to livestock and humans [23,24,25,26,27]. Research has shown that many natural compounds can alleviate the reproductive toxicity of ZEN through antioxidant effects. However, the efficacy of each drug varies for different species and new antagonistic drugs are constantly being discovered [18]. In this study, nine natural compounds were identified as potential therapeutic drugs based on a natural compound library and previous transcriptome results. Based on the expression of oocyte maturation genes, three antioxidant drugs, oxysophoridine, rutin, and phellodendrine, were found to reduce the negative effects of ZEN. By investigating concentration gradients, the optimal concentrations for oxysophoridine, rutin, and phellodendrine were determined to be 10^−6^, 10^−8^, and 10^−7^ mol/L, respectively, indicating their different therapeutic effects for ovines.

To understand the mechanisms of action of the three drugs, their effects on the expression of relevant genes related to ovine oocyte maturation, spindle checkpoint, cell cycle, cumulus expansion factor, oxidative stress, autophagy, and apoptosis were analyzed. The results showed the following: (1) ZEN inhibited the expression of genes related to oocyte maturation (*BMP15*, *CDC20*, and *GDF9*), cell cycle (*CDK1* and *CyclinB1*), and spindle assembly checkpoints (*BUB1* and *MAD2L1*). All three drugs promoted the expression of these genes, restoring or even surpassing their original expression levels. Oxysophoridine yielded the best effect, followed by rutin and phellodendrine; (2) ZEN inhibited the expression of genes related to the follicle expansion factor (*HAS2*, *PTGS2*, and *TNFAIP6*), while oxysophoridine and phellodendrine promoted the expression of these three genes, returning to normal or higher levels. Rutin only promoted the expression of *HAS2* and *TNFAIP6*; (3) ZEN inhibited the expression of genes related to oxidative stress (*GPX*, *SOD1*, and *SOD2*) and all three drugs promoted the expression of *SOD1* and *SOD2*. Oxysophoridine promoted the expression of *GPX*, while the other two drugs had inhibitory effects; (4) ZEN inhibited the expression of *LC3*, whereas all three drugs promoted the expression of this gene. ZEN promoted the expression of *ATG3* and *ULK2*, while oxysophoridine inhibited the expression of *ATG3* and promoted the expression of *ULK2*. Opposite results were obtained for rutin and phellodendrine, indicating that the three drugs affect autophagy differently, which may determine whether autophagy is activated by the *LC3* ubiquitin-like binding system or the *ULK1* kinase core complex; and (5) ZEN promoted the expression of *CAS3*, *CAS8*, *CAS9*, and *P53*, while it inhibited the expression of *GPX*, indicating that it promotes cell apoptosis. The three drugs exhibited contrasting effects to ZEN, inhibiting apoptosis.

Based on these results, the mechanisms of action of the three drugs can be inferred (Figure 10B). The reproductive toxicity of ZEN to in vitro ovine oocyte maturation can be reduced through antioxidant, pro-autophagic, and anti-apoptotic mechanisms; however, the specific mechanisms of action differ. Oxysophoridine promotes *GPX*, *SOD1*, *SOD2*, *ULK2*, and *LC3* expression and inhibits *ATG3*, *CAS3* and *CAS8* expression; these results are similar to reports stating that it improved spinal cord and myocardial injuries by increasing *SOD* expression and reducing the expression of inflammatory factors (e.g., *IL*-6, *IL*-8) and *CAS3* [28,29]. Rutin promoted *SOD2*, *LC3* and *BAX*, and inhibited *ULK2*, *CAS3* and *CAS9*, which is consistent with reports stating that it enhances the expression of antioxidant enzymes (e.g., *SOD*, *CAT*, *GPX*) and inhibits the expression of apoptotic genes such as *CAS3*, *CAS7*, *CAS9*, and *P53* [26,30]. Phellodendrine promoted *SOD1*, *SOD2*, *ATG3* and *LC3*, and inhibited *CAS3* and *CAS9*, which is consistent with reports of it regulating the AMPK/mTOR pathway, reducing *PTGS1*, *PTGS2*, *AKT* phosphorylation, and NF-kB3, and having autophagy, anti-inflammatory, and antioxidant effects [31,32,33]. All three compounds promoted the expression of oxidative stress-related genes, reduced the accumulation of intracellular ROS, inhibited the expression of apoptotic genes, reduced mitochondrial damage, promoted polar body emissions, and inhibited cell apoptosis. Furthermore, the compounds affected genes in a way that could promote autophagy (LC3 ubiquitin-like binding system or ULK1 kinase core complex system), degradation of organelle fragments within cells, expression of cell cycle and spindle checkpoint genes, oocyte maturation, and release of cumulus expansion factors (Figure 10B). Through such alterations in gene expression, the compounds antagonize the reproductive toxicity of ZEN in ovine oocytes and promote their maturation (Figure 10B). However, further research on the specific genes through which the three drugs exert their effects, and their subsequent applications in sheep farming, are required.

## 5. Conclusions

In summary, six antioxidant- and three autophagy-related compounds were preliminarily identified in the present study based on previous Smart-seq2 results of inhibition of in vitro maturation of ovine oocytes using ZEN. The preliminary analysis showed that antioxidant drugs more effectively antagonize the reproductive toxicity of ZEN in ovine oocytes. According to the qPCR results related to oocyte maturation, cumulus expansion factors, spindle assembly checkpoints, and cell cycle-related genes, the positive effects of the three tested natural compounds can be ranked in descending order, as follows: oxysophoridine, rutin, and phellodendrine. All three compounds reduced intracellular ROS accumulation, inhibited cell apoptosis, and enhanced autophagy, thereby stabilizing the intracellular environment, maintaining normal cell cycle, and promoting oocyte maturation and the release of cumulus expansion factors, ultimately antagonizing ZEN reproductive toxicity on ovine oocytes and promoting in vitro maturation of ovine oocytes. In the present study, three natural compounds that effectively antagonized the reproductive toxicity of ZEN in ovine oocytes were identified and the underlying mechanisms were elucidated and compared. This work serves as a reference and theoretical support for studies on the reproductive toxicity mechanisms of ZEN, application of the three identified compounds in the ovine breeding industry, improvement of the mechanism of action of the three compounds, and protection of human reproductive health.

## Figures and Tables

**Table 1:** All gene primer information for qPCR.

Primers	Primer Sequences	Product Length (bp)	Annealing Temp (°C)	Gene Accession Number
*BMP15*	GGACACCCTAGGGAAAACCG	101	60	NM_001114767.2
	TGTATGTGCCAGGAGCCTCT			
*CDC20*	GGCTGAGCTGAAAGGTCACA	214	60	XM_004023553.6
	AACACCGTGAGGAGTTGGTC			
*GDF9*	TGACAGAGCTTTGCGCTACA	166	61	NM_001142888.2
	TGATGGAAAGGTTCCTGCCG			
*HAS2*	GGAGACATATCGCTGCTGCT	217	61	XM_004011666.5
	ACCCACATAAAGCATGGCTAGT			
*PTGS2*	GACCATGGTAGAAGCCGGAG	294	61	NM_001009432.1
	AGTTCGGTTGAACGCTCCTT			
*TNFAIP6*	GCTATGGGAAGAGGCTCACG	149	61	NM_001009432.1
	ATTCACACACTGCCTTCGCT			
*CDK1*	ATGGCTTGGATCTGCTCTCGAA	154	61	NM_001142508.1
	TGCTCTTGACACAACACAGGA			
*CyclinB1*	GCTTGGAGACATCGGTAACA	129	60	XM_060414226.1
	GGAGCCTTTTCCAGAGGTTTTG			
*BUB1*	ACGCCTCACTGAAACCCATT	195	61	XM_012173898.5
	GTGATCACCCTTTGTTCCCCT			
*MAD2L1*	CCTTTTGAAACGAGTGGCGG	167	60	XM_004009585.5
	GAGAAGAACTCGGCCACGAT			
*GPX*	CAGTTTGGGCATCAGGAAAAC	100	61	XM_004018462.5
	CGAAGAGCATGAAATTGGGC			
*SOD1*	GGCAATGTGAAGGCTGACAA	130	61	NM_001145185.2
	TGCCCAAGTCATCTGGTCTT			
*SOD2*	GGACAAATCTGAGCCCCAAC	180	61	NM_001280703.1
	CAATCTGTAAGCGTCCCTGC			
*ATG3*	CCCGGTCCTCAAGGAATCAA	103	61	XM_004002919.6
	TTGCCATGTTGGACAGTGGT			
*LC3*	ACGCCTCTCAGGAGACTTTTG	121	61	XM_004014953.4
	ACCTCAGTTGGTAACATCCCT			
*ULK2*	GTGAAGCAAGGTTCAAGCCG	132	61	XM_060395488.1
	TCAATGTCTGCTGGGTCCTG			
*BAX*	TTCCGACGGCAACTTCAACT	260	61	XM_027978594.3
	CCATGTGGGTGTCCCAAAGT			
*CAS3*	ACGGAAGCAAATCAGTGGAC	167	61	XM_060406953.1
	GGTTTCCCTGAGGTTTGCTG			
*CAS8*	AGTGAGTTGCAGACATCCGA	172	61	XM_060410232.1
	AGGTCTTGTCCAAAGCCTCT			
*CAS9*	AGAGTGATGAAGCAGGACCC	195	61	XM_060396599.1
	CAGATCGGCATTTCCCTTGG			
*P53*	TCTTCAGATCCGTGGGCGTA	158	61	NM_001009403.1
	TTTTATGGCAGGAGGGAGAAGG			
*GAPDH*	AGATGGTGAAGGTCGGAGTG	188	60	XM_060411595.1
	GTTCTCTGCCTTGACTGTGC			

**Table 2:** During the initial drug screening period, information for each group, number of trials, number of oocytes in each trial, and maturation rate.

Group	First Time		Second Time		Third Time		Maturation Rate/%
	Initial Quantity (pcs)	Sample Quantity Received (pcs)	Initial Quantity (pcs)	Sample Quantity Received (pcs)	Initial Quantity (pcs)	Sample Quantity Received (pcs)	
CK	17	14	16	14	17	15	51.26984 ± 5.35229
ZEN (20 μmol/L)	17	15	16	14	17	15	29.68254 ± 5.22365
ZEN (20 μmol/L) + Diosgenin glucoside (10^−6^ mol/L)	17	15	16	15	17	14	27.14286 ± 5.96665
ZEN (20 μmol/L) + Syringin (10^−6^ mol/L)	17	15	16	13	17	15	46.83761 * ± 6.92466
ZEN (20 μmol/L) + Liensinine (10^−6^ mol/L)	17	16	16	14	17	16	32.44048 ± 4.58179
ZEN (20 μmol/L) + Phellodendrine (10^−6^ mol/L)	17	14	16	15	17	15	50.15873 ** ± 6.04843
ZEN (20 μmol/L) + Oxysophoridine (10^−6^ mol/L)	17	15	16	14	17	14	55.71429 ** ± 5.15079
ZEN (20 μmol/L) + Isolongifolene (10^−6^ mol/L)	17	15	16	14	17	13	45.22589 * ± 2.06735
ZEN (20 μmol/L) + L-Ascorbic acid (10^−6^ mol/L)	17	14	16	15	17	15	45.55556 * ± 5.09175
ZEN (20 μmol/L) + Morroniside (10^−6^ mol/L)	18	15	16	14	17	15	47.61905 * ± 5.30263
ZEN (20 μmol/L) + Rutin (10^−6^ mol/L)	18	16	18	16	19	16	47.91667 * ± 3.60844

**, *p* < 0.01 is extremely significant; *, *p* < 0.05 is significant.

**Table 3:** Information of each group during working concentration screening.

Drug Group	Grouping of Each Drug	First Time		Second Time		Third Time		Maturation Rate/%
		Initial Quantity (pcs)	Sample Quantity Received (pcs)	Initial Quantity (pcs)	Sample Quantity Received (pcs)	Initial Quantity (pcs)	Sample Quantity Received (pcs)	
Syringin	CK	25	20	21	17	20	16	49.4363 ± 5.98998
	ZEN (20 μmol/L)	25	23	23	21	20	18	31.9646 ± 3.69757
	ZEN (20 μmol/L) + Syringin (10^−4^ mol/L)	25	24	23	19	20	15	29.8068 ± 3.62705
	ZEN (20 μmol/L) + Syringin (10^−5^ mol/L)	25	20	23	20	20	17	36.7647 ± 2.80570
	ZEN (20 μmol/L) + Syringin (10^−6^ mol/L)	25	22	23	22	20	16	38.8258 ± 6.23276
	ZEN (20 μmol/L) + Syringin (10^−7^ mol/L)	25	21	23	18	20	17	34.2515 ± 5.23716
	ZEN (20 μmol/L) + Syringin (10^−8^ mol/L)	32	30	23	22	23	20	28.6869 ± 5.42359
Oxysophoridine	CK	19	17	22	18	20	18	49.0196 ± 5.80928
	ZEN (20 μmol/L)	19	18	22	19	20	18	34.6004 ± 3.81613
	ZEN (20 μmol/L) + Oxysophoridine (10^−4^ mol/L)	19	15	22	17	20	16	35.6373 ± 5.53444
	ZEN (20 μmol/L) + Oxysophoridine (10^−5^ mol/L)	19	17	22	22	20	17	48.4848 ** ± 3.94177
	ZEN (20 μmol/L) + Oxysophoridine (10^−6^ mol/L)	19	17	22	16	20	17	51.9608 ** ± 1.69809
	ZEN (20 μmol/L) + Oxysophoridine (10^−7^ mol/L)	19	17	22	20	20	15	46.2418 * ± 1.09318
	ZEN (20 μmol/L) + Oxysophoridine (10^−8^ mol/L)	17	12	20	15	22	19	39.5029 ± 2.45039
Rutin	CK	22	20	18	16	23	20	45.0000 ± 5.00000
	ZEN (20 μmol/L)	22	20	18	15	23	21	28.9683 ± 4.18081
	ZEN (20 μmol/L) + Rutin (10^−4^ mol/L)	22	16	20	17	23	18	35.2533 ± 5.23434
	ZEN (20 μmol/L) + Rutin (10^−5^ mol/L)	22	21	20	16	23	19	35.3958 ± 3.64461
	ZEN (20 μmol/L) + Rutin (10^−6^ mol/L)	22	17	20	20	23	20	34.8039 ± 5.29684
	ZEN (20 μmol/L) + Rutin (10^−7^ mol/L)	22	17	20	17	23	17	45.0980 ** ± 3.39618
	ZEN (20 μmol/L) + Rutin (10^−8^ mol/L)	20	18	20	16	22	19	48.7939 ** ± 4.56198
Phellodendrine	CK	25	18	24	20	18	16	50.1852 ± 5.28021
	ZEN (20 μmol/L)	25	18	24	22	18	14	31.7701 ± 3.96847
	ZEN (20 μmol/L) + Phellodendrine (10^−4^ mol/L)	31	29	24	19	18	17	30.3370 ± 5.68084
	ZEN (20 μmol/L) + Phellodendrine (10^−5^ mol/L)	25	25	24	20	18	16	32.7500 ± 1.98431
	ZEN (20 μmol/L) + Phellodendrine (10^−6^ mol/L)	25	21	24	22	18	15	39.7403 ± 3.25454
	ZEN (20 μmol/L) + Phellodendrine (10^−7^ mol/L)	25	18	24	21	18	16	52.6455 ** ± 2.78721
	ZEN (20 μmol/L) + Phellodendrine (10^−8^ mol/L)	25	19	22	19	22	19	43.8596 ± 6.07737

**, *p* < 0.01 is extremely significant; *, *p* < 0.05 is significant.

**Table 4:** Three types of drug trial information.

Group	First Time		Second Time		Third Time		Fourth Time		Maturation Rate/%
	Initial Quantity (pcs)	Sample Quantity Received (pcs)	Initial Quantity (pcs)	Sample Quantity Received (pcs)	Initial Quantity (pcs)	Sample Quantity Received (pcs)	Initial Quantity (pcs)	Sample Quantity Received (pcs)	
CK	20	16	21	19	23	17	21	17	49.0954 ± 4.47271
ZEN (20 μmol/L)	20	17	21	15	23	17	21	17	28.9216 ± 6.27757
ZEN (20 μmol/L) + Oxysophoridine (10^−6^ mol/L)	20	17	21	19	23	18	21	16	54.3446 ** ± 1.82583
ZEN (20 μmol/L) + Rutin (10^−8^ mol/L)	20	18	21	17	23	19	21	19	50.5805 ** ± 5.05156
ZEN (20 μmol/L) + Phellodendrine (10^−7^ mol/L)	20	13	21	19	23	18	23	18	51.6925 ** ± 3.70242

**, *p* < 0.01 is extremely significant.

## Data Availability

The data that support the findings of this study are available from the corresponding author on reasonable request.
